# Effectiveness of magnetic seizure therapy versus electroconvulsive therapy for major depressive episode in China: protocol for a double-blind, randomized, non-inferiority trial

**DOI:** 10.3389/fpsyt.2025.1657906

**Published:** 2025-10-23

**Authors:** TianHong Zhang, YongGuang He, Sha Liu, MingLiang Ju, YiYi Yang, RunChun Zhou, YaWen Hong, YanYan Wei, XiaoChen Tang, HaoYang Zheng, YuPing Jia, HaiChun Liu, DongSheng Zhou, Qiang Hu, Ran Wang, Wei Zheng, LingYun Zeng, CuiXia An, ChunBo Li, JiJun Wang

**Affiliations:** ^1^ Shanghai Mental Health Center, Shanghai Jiaotong University School of Medicine, Neuromodulation Center, Shanghai Engineering Research Center of Intelligent Psychological Evaluation and Intervention, Shanghai Key Laboratory of Psychotic Disorders, Shanghai, China; ^2^ Department of Psychiatry, First Hospital/First Clinical Medical College of Shanxi Medical University, Taiyuan, China; ^3^ Department of Automation, Shanghai Jiao Tong University, Shanghai, China; ^4^ Department of Psychiatry, Ningbo Kangning Hospital, Ningbo, China; ^5^ Department of Psychiatry, Wuhu Hospital of Anding Hospital (The Fourth People’s Hospital of Wuhu), Anhui, China; ^6^ Department of Psychiatry, The First Hospital of Hebei Medical University, Shijiazhuang, Hebei,, China; ^7^ The Affiliated Brain Hospital of Guangzhou Medical University, Guangzhou, China; ^8^ Department of Psychiatric Rehabilitation, Shenzhen Kangning Hospital, Shenzhen, China

**Keywords:** seizure treatment, electroconvulsive therapy, magnetic seizure therapy, randomized controlled trial, major depressive disorder

## Abstract

Electroconvulsive therapy (ECT) has been a cornerstone in treating Major Depressive Disorder (MDD), demonstrating high efficacy but often limited by cognitive side effects. As an alternative, magnetic seizure therapy (MST) has emerged, offering comparable antidepressant effects with potentially fewer cognitive adverse outcomes. This study aimed to compare the efficacy, safety, and cognitive impact of MST and ECT. This multicenter, double-blind, parallel, non-inferiority randomized clinical trial will be conducted at seven clinical centers. Participants diagnosed with MDD will be randomly allocated to either the ECT or MST group. Each center aims to recruit 30 participants, resulting in a total sample size of 210. The intervention includes 12 sessions over 4 weeks, followed by 12-week follow-up. Assessments (the 24-item Hamilton Depression Rating Scale (HDRS-24), Hamilton Anxiety Scale (HAMA), 8 cognitive tests, Electroencephalography, electrocardiography) occur at baseline, post-sessions 3/6/9/12 (3 hours after each session), and 4-/8-/12-week follow-ups. The primary outcome is the change in HDRS-24 total score from baseline to the 12-week follow-up, which is assessed after the completion of the 12-session acute treatment phase (conducted over 4 weeks) and a subsequent 12-week post-treatment observation period. Randomization and blinding protocols will be strictly followed to ensure unbiased treatment administration and assessment. This trial aims to provide robust evidence that MST is as effective as ECT in reducing depressive symptoms (assessed via HDRS-24) and to confirm its superior cognitive safety, with the Hopkins Verbal Learning Test-Revised as an additional primary outcome to evaluate verbal memory changes. Remaining cognitive measures will serve as secondary outcomes. Exploratory analyses will examine electroencephalography and electrocardiography data to identify potential neurophysiological biomarkers. If successful, this trial could significantly influence clinical practices and improve seizure treatment for patients with MDD.

**Clinical trial registration:**

ClinicalTrials.gov, identifier NCT06409325.

## Introduction

Electroconvulsive therapy (ECT), introduced in 1938, has long been a cornerstone treatment for mood disorders, notably Major Depressive Disorder (MDD) and treatment-resistant depression (1). Administered under anesthesia, ECT involves delivering controlled electrical currents to the scalp to induce a therapeutic seizure. Despite its efficacy, with remission rates ranging from 50% to 70% (2), ECT is underutilized due to various factors including fear, stigma, and concerns about cognitive side effects, such as short-term amnesia (3).

In pursuit of alternative treatments with comparable efficacy but fewer side effects, researchers have explored technique such as magnetic seizure therapy (MST). The first documented use of MST in humans (4) followed closely after its initial application in animal studies (5). Subsequent clinical trials have primarily focused on assessing the feasibility, efficacy, and safety of MST as a treatment for depression. Lisanby and colleagues provided early evidence supporting the feasibility of MST for depression and reported no serious adverse events associated with its use (6). Kayser and colleagues observed antidepressant effects of MST comparable to those of ECT (7). Fitzgerald and colleagues further asserted the antidepressant effects of MST, noting a lack of apparent cognitive adverse effects (8).

### Comparative action mechanisms of ECT vs. MST

Both ECT and MST utilize the induction of a seizure to achieve therapeutic effects. However, they differ significantly in the methods through which this seizure is induced and the resulting neurobiological impact (9). ECT achieves seizure induction through high-frequency, repetitive transcranial electrical stimulation. In contrast, MST employs high-frequency, repetitive transcranial magnetic stimulation. This distinction is critical as the method of stimulation impacts the nature and spread of the induced seizure. With MST, a rapid, high-intensity, time-varying magnetic field is applied to the brain, bypassing resistance and allowing for targeted stimulation of specific neurons based on the geometry of the stimulating magnetic coil. Unlike ECT, the magnetic field in MST is not impeded by non-conducting material, such as the skull, resulting in more focal brain activation (10, 11). A computational study by Deng et al. (12). compared the electric field induced by ECT and MST paradigms. They found that ECT induced a maximum electric field in the brain of 2.1–2.5 V/cm, while MST induced a field of 1.1–2.2 V/cm. This corresponds to 6.2–7.2 times and 1.2–2.3 times the neural activation threshold, respectively. Notably, the electric field induced by MST is more confined to the superficial cortex compared to ECT. The volume of the brain stimulated is significantly higher with ECT (up to 100%) compared to MST (up to 8.2%). Among MST techniques, the double cone coil exhibits the most focal stimulation, whereas bilateral ECT is the least focal. Specifically, compared to right unilateral ultra-brief pulse ECT, the electric field induced by MST is 5–10 times more focal (12, 13). While both ECT and MST achieve therapeutic effects through seizure induction, their distinct mechanisms of action result in differences in spatial specificity and depth of stimulation. MST, with its more focal and targeted approach, offers a promising alternative to ECT with potentially fewer side effects and greater therapeutic precision.

### Comparative efficacy of ECT vs. MST

Previous head-to-head studies comparing the antidepressant efficacy of ECT and MST have generally found comparable treatment outcomes (14). However, recent research has provided further insights into the effectiveness of these therapies through controlled clinical trials and systematic reviews. A double-blinded, randomized clinical trial conducted by Deng et al. (15). compared the antidepressant effects of MST, applied at 100 Hz for 10 seconds at 100% of the maximum device power, with ultrabrief pulse right unilateral ECT, applied at 6 times seizure threshold. Both MST and ECT demonstrated clinically meaningful antidepressant effects, with similar response and remission rates observed in both treatment groups. Additionally, an open-label study by Daskalakis et al. (16). evaluated the efficacy of MST at different frequencies (i.e., high-frequency MST at 100 Hz, medium-frequency MST at 60 or 50 Hz, and low-frequency MST at 25 Hz) using 100% stimulator output. Among patients with MDD, high-frequency MST produced the highest remission rate, indicating its potential as an effective treatment option. In a study by Kayser et al. (7), twenty patients with treatment-resistant depression were randomly assigned to receive either MST or ECT. The study found that both treatments produced a statistically significant and similar improvement in depressive symptoms, as measured by a 50% reduction in Montgomery Åsberg Depression Scale ratings. Several systematic reviews have also examined the comparative efficacy of MST versus ECT for depression treatment. Cai et al. (17). summarized four randomized controlled trials (RCTs) and found no significant differences in response, remission, or improvement in depressive symptoms between MST and ECT. Similarly, a meta-analysis by Chen et al. (18). included data from 10 studies comprising a total of 285 patients. The analysis revealed no significant difference in the antidepressant effect between MST and ECT, further supporting the equivalence of these treatments in depression management.

### Comparative side-effect of ECT vs. MST

The comparison of cognitive side effects between ECT and MST in the treatment of MDD has yielded varying conclusions. However, the majority of studies suggest that MST results in fewer physical side effects and no cognitive impairments compared to ECT. In a RCT by Deng et al. (15), patients receiving ECT reported higher severity of headache, nausea, and muscle pain compared to those receiving MST. Furthermore, patients receiving ECT experienced greater confusion or disorientation, and took significantly longer to regain orientation compared to MST (19). Autobiographical recall is linked to temporal cortices, notably the hippocampus. This suggests that ECT may exert a more detrimental effect on the hippocampus compared to MST. This supports the cognitive safety of MST, as evidenced by negligible cognitive adverse effects and superior performance in autobiographical memory recall. A systematic review (20) focusing on the cognitive effects of MST found little to no adverse cognitive effects. While some RCTs comparing MST and ECT reported inconsistent results regarding cognitive effects, the accumulated evidence indicates that MST has fewer adverse cognitive effects compared to ECT (17). A pilot study by Fitzgerald et al. (21) compared the cognitive effects of 100 Hz MST and ECT in patients with persistent depression. Significant improvements in psychomotor speed, verbal memory, and cognitive inhibition were observed following MST, with no reductions in cognitive performance. Conversely, ECT resulted in significant improvement in only one cognitive inhibition task. Furthermore, the MST group showed significantly greater improvement in psychomotor speed compared to ECT. In an open-label study by Kayser et al. (7), MST was investigated as an add-on therapy to controlled pharmacotherapy for treatment-resistant depression (TRD). No cognitive side effects were observed in either group, suggesting the cognitive safety of MST. Polster et al. (22) aimed to broaden insight into the side effect profile of MST compared to ECT by examining the disruption of acute verbal memory processes after treatment. Overall, evidence from clinical trials and systematic reviews suggests that MST is associated with fewer physical side effects and superior cognitive safety compared to ECT in the treatment of MDD.

### Biomarker rationale

Neurophysiological biomarkers offer a unique opportunity to tailor seizure therapies to individual patients. For instance, EEG can capture real-time seizure characteristics and brain connectivity, while ECG HRV reflects autonomic nervous system dynamics-both of which are disrupted in MDD and modulated by MST/ECT (23, 24). By linking these biomarkers to treatment outcomes, we aim to establish a data-driven framework for personalized therapy, addressing the heterogeneity of MDD and optimizing treatment selection. This approach builds on prior work showing that MST’s focal stimulation elicits distinct EEG signatures compared to ECT (25), and that HRV abnormalities predict treatment response and side effects.

### Study objectives

Despite advancements in MST and ECT for MDD, there remains a need for further research to replicate clinical outcomes and understand the therapeutic targets of these treatments. This project titled “Chinese Union: Replication, Efficacy, and Safety of Seizure Therapy with Evaluation-based Precision (CURES-STEP)” phase I initiated by the [removed for peer review], aims to address these gaps through a double-blinded, randomized, non-inferiority investigation comparing the efficacy, tolerability, cognitive adverse effects, and neurophysiological biomarkers of MST and bilateral ECT in patients with MDD. The primary objectives of the study are twofold: 1. To replicate the comparable antidepressant efficacy and reduced side effects (with HVLT-R as an additional primary outcome) observed in previous comparisons between MST and ECT. 2. To utilize electroencephalography (EEG) (25) and electrocardiography (ECG) (23) markers as neurophysiological biomarkers to assist in treatment decision-making between ECT and MST, and to predict treatment outcomes including antidepressant effects and cognitive side effects. We believe that MST and ECT are not simply interchangeable treatments, and that leveraging neurophysiological characteristics may help tailor treatment decisions for individual patients with MDD. By examining both clinical outcomes and neurophysiological biomarkers, we aim to provide valuable insights into the efficacy, safety, and personalized treatment approaches for MDD using seizure therapies. Additionally, this study aims to validate the clinical application value of a domestically developed MST device approved by the National Medical Products Administration (NMPA) in China, as previous MST studies utilized the MagPro MST (MagVenture A/S, Denmark) device (26, 27).

## Methods

### Study settings

This multicenter trial will be conducted at seven clinical center: Shanghai Mental Health Center (SMHC), First Hospital of Shanxi Medical University, The Affiliated Brain Hospital of Guangzhou Medical University, The First Hospital of Hebei Medical University, Shenzhen Kangning Hospital, Ningbo Kangning Hospital, The Fourth People’s Hospital of Wuhu. All interventions and assessments will take place in the neuromodulation department of these hospitals. EEG and ECG acquisitions will be conducted in the electrophysiology department within the same clinical institution. The trial was commenced from December 1, 2024 and conclude on December 30, 2027. The authors assert that all procedures contributing to this work comply with the ethical standards of the relevant national and institutional committees on human experimentation and with the Helsinki Declaration of 1975, as revised in 2013. All procedures involving human subjects/patients were approved by the Ethics Committee of SMHC (Approval No. 2025-21) and registered on the clinical trials website prior to the commencement of enrollment (NCT06409325, ClinicalTrials.gov).

### Study design

The present clinical protocol is designed as a double-blind, parallel, non-inferiority, randomized clinical trial.

### Study summary

**Figure 1:**
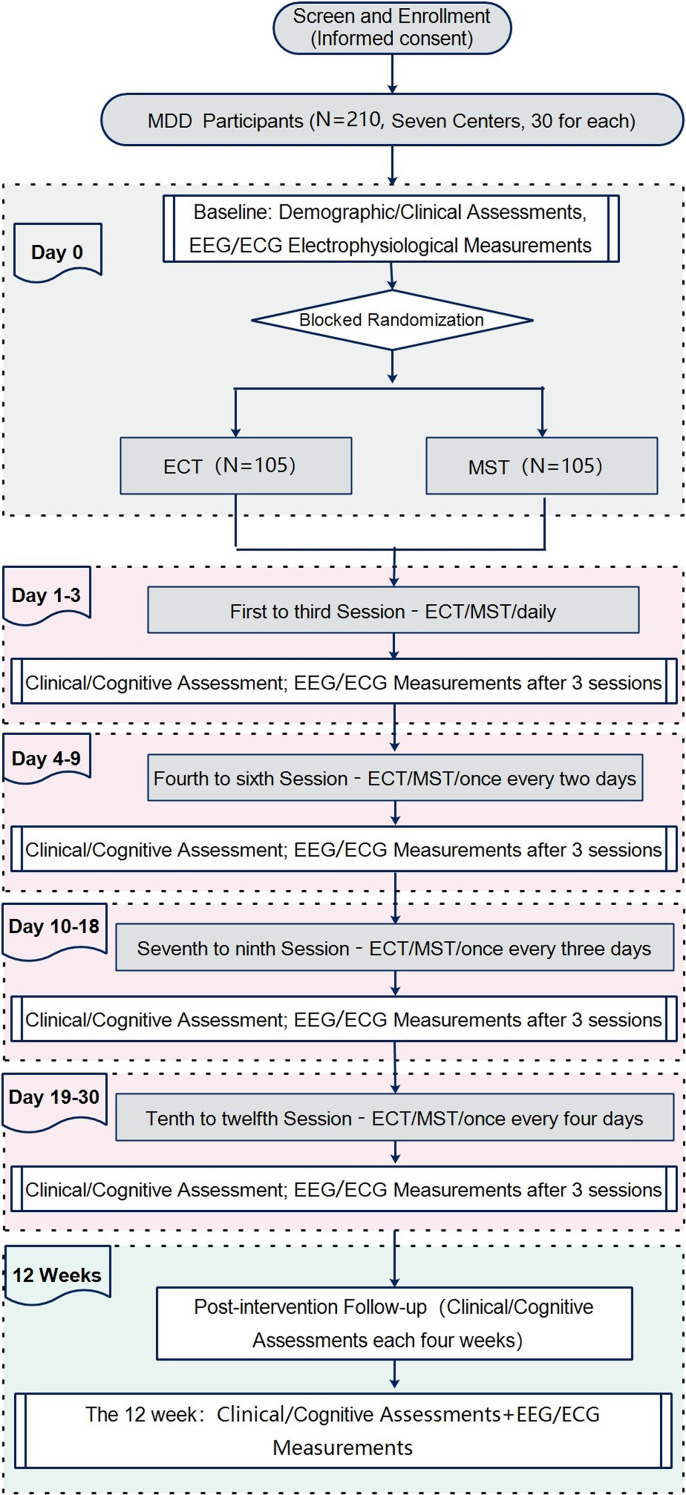
Flow diagram of the study design. This flowchart outlines the trial design comparing electroconvulsive therapy (ECT) and magnetic seizure therapy (MST) in patients with major depressive disorder (MDD). A total of 210 MDD participants (recruited from 7 centers, 30 per center) undergo screening and enrollment with informed consent. At baseline (Day 0), demographic/clinical assessments and electrophysiological measurements (EEG/ECG) are conducted. Participants are then randomized via blocked randomization to either the ECT group (n=105) or MST group (n=105). Treatment sessions are delivered in four phases: Days 1–3 (sessions 1–3, daily), Days 4–9 (sessions 4–6, every 2 days), Days 10–18 (sessions 7–9, every 3 days), and Days 19–30 (sessions 10–12, every 4 days). Clinical/cognitive assessments and EEG/ECG measurements are performed after every 3 sessions (end of each phase). A 12-week post-intervention follow-up includes clinical/cognitive assessments every 4 weeks, with additional EEG/ECG measurements at week 12.

**Figure 1** provides an overview of the study flow. Participants will be randomly allocated to either the ECT or MST group. Each center aims to recruit 15 participants for each group (ECT and MST), using block randomization, resulting in a total of 30 participants per center (15 per group). Across seven centers, this will yield a total sample size of 210 participants (105 per group). The trial comprises a 12-session intervention phase of ECT/MST, spanning approximately 4 weeks, followed by a 12-week observation period. For the first three treatment sessions, participants will receive consecutive sessions. Subsequently, there will be a one-day interval between sessions 4 to 6, a two-day interval between sessions 7 to 9, and a three-day interval between sessions 10 to 12, ensuring completion within a month. Following treatment completion, participants will undergo follow-up clinical observations every four weeks for 12 weeks. EEG and ECG recordings will be obtained at baseline, post-session 3, 6, 9, 12 (3 hours after each session), and at the 12-week follow-up. All evaluations will be conducted under standardized conditions throughout the sessions. **Table 1** delineates the time points for clinical assessments, interventions, and electrophysiological examinations.

**Table 1:** Schematic summary and milestones for enrolment, ECT/MST treatment, and assessments across the study time-line.

Time-point	Eligibility screen	Informed consent	Allocation	Intervention (ECT/MST)	Assessments			
					Demographic features	Clinical /Cognitive performances	EEG	ECG
Day 0	X	X	X		X	X	X	X
Treatment phase								
Day 1				X				
Day 2				X				
Day 3				X		X	X	X
Day 5				X				
Day 7				X				
Day 9				X		X	X	X
Day 12				X				
Day 15				X				
Day 18				X		X	X	X
Day 22				X				
Day 26				X				
Day 30				X		X	X	X
Post-treatment phase								
Week 4						X		
Week 8						X		
Week 12						X	X	X

### Participants and recruitment

The recruitment process will target patients from seven participating centers. Eligibility assessment will exclusively involve individuals receiving clinical services at these facilities. Initial screening of potential participants meeting the eligibility criteria will be conducted by two experienced licensed psychiatrists at each center, each with a minimum of five years of clinical experience. Upon confirming eligibility, detailed information about the protocol will be provided to the participants and their legal guardians. Both participants and their legal guardians must sign a consent form indicating their willingness to participate in the study before official enrollment.

### Inclusion criteria

1. Participants must meet the diagnostic criteria for MDD as outlined in the Diagnostic and Statistical Manual of Mental Disorders, Fifth Edition (DSM-5).
2. Participants must have a baseline score of 18 or higher on the 24-item Hamilton Depression Rating Scale (HDRS-24) (28).
3. All patients are MST/ECT naïve and are required to be considered suitable for a course of ECT by both their treating psychiatrist and the study psychiatrist.
4. During the trial’s treatment period, participants must be using a single antidepressant medication at a stable dose.
5. Participants must be between the ages of 18 and 65 years.
6. Informed consent from both parents and legal guardians is required.

### Exclusion criteria

1. Individuals with current or history of organic brain disorders or neurological disorders will be excluded from the study.
2. Participants with a Wechsler Abbreviated Scale of Intelligence (WASI) score of less than 70 will be excluded.
3. Presence of contraindications to anesthesia, such as: allergic reactions to anesthesia medications.
4. Individuals currently taking antiepileptic drugs, benzodiazepines, or other medications that may affect seizure activity will be excluded from the study.
5. Those with exposure to ECT, modified ECT, MST, transcranial magnetic stimulation (TMS), transcranial direct current stimulation (tDCS), transcranial alternating current stimulation (tACS), or other neurostimulation treatments in last 6 months will be excluded.
6. Individuals with cochlear implants, cardiac pacemakers, implanted devices, or metal in the brain will be excluded from participation.
7. Pregnant or lactating individuals will be excluded from the study.
8. Individuals currently participating in another concurrent clinical trial will not be eligible for inclusion.
9. Participants who refuse to provide informed consent to participate in the trial will be excluded.
10. Other circumstances deemed unsuitable for participation by researchers will result in exclusion.

### Sample size estimation

**Figure 2:**
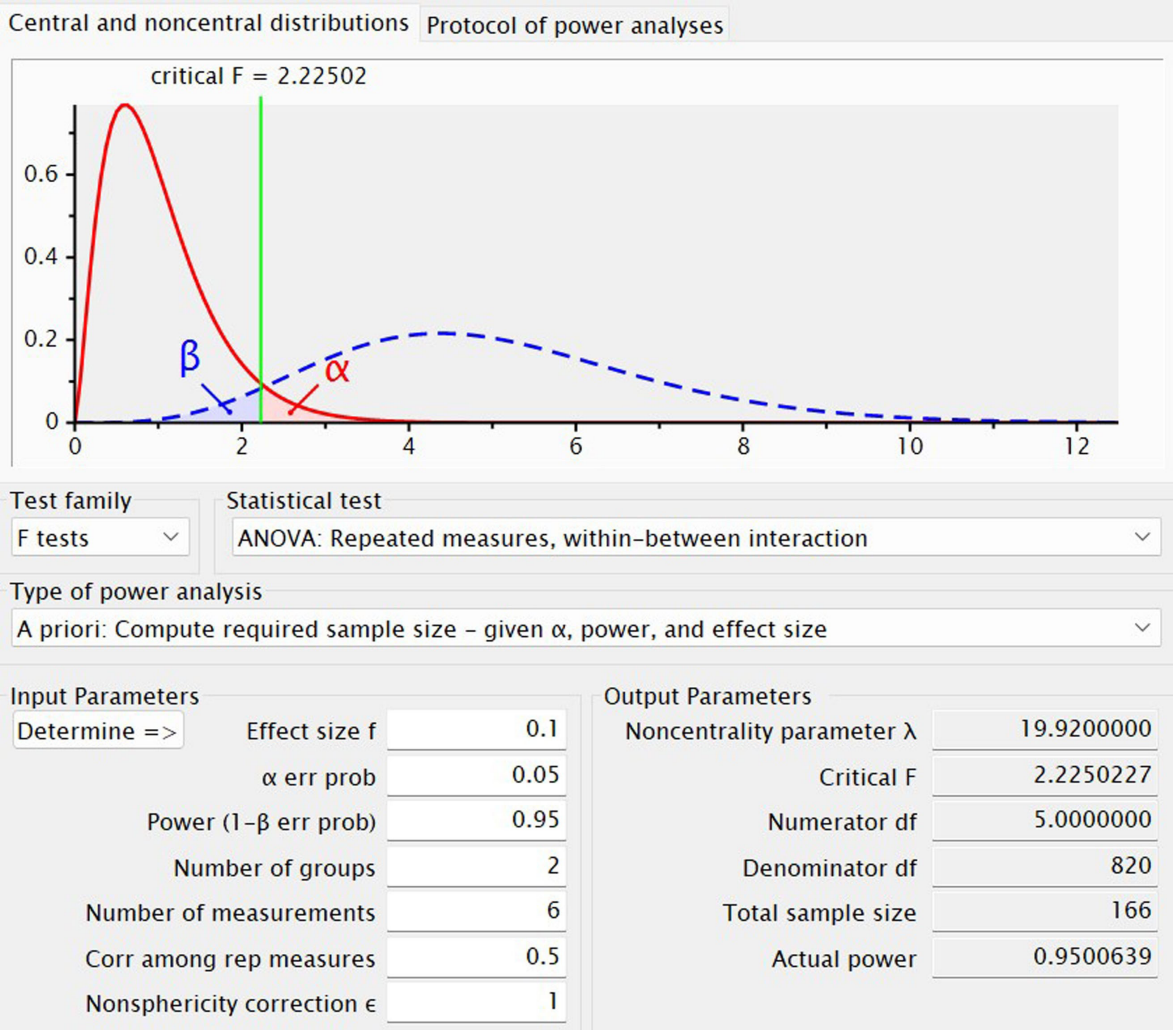
G*Power-based sample size calculation for a repeated-measures ANOVA with a within-between interaction. The plot depicts an *a priori* power analysis to determine the required sample size for a repeated-measures analysis of variance examining the interaction between a between-subjects factor (2 groups) and a within-subjects factor (6 measurement time points). The upper panel shows the central F-distribution under the null hypothesis (red solid curve) and the noncentral F-distribution under the alternative hypothesis (blue dashed curve). The critical F-value (2.22502) is indicated by the green vertical line. The red shaded region represents the α error probability (Type I error, set at 0.05), and the blue shaded region denotes the β error probability (Type II error), with power (1−β) targeted at 0.95. Input parameters include: effect size (f) = 0.1, α = 0.05, number of groups = 2, number of repeated measurements = 6, correlation among repeated measures = 0.5, and sphericity correction (ϵ) = 1. Output parameters reveal a total required sample size of 166, a noncentrality parameter (λ) of 19.92, numerator degrees of freedom (df) = 5, denominator df = 820, and an actual power of ~0.95.

The sample size calculation for this study was conducted through *a priori* power analysis using G*Power 3.1.9.7 (29). Parameters were chosen, including α = 0.05 and power (1-ß) = 0.95. An effect size of f = 0.1 was selected based on previous research findings (15). Specifically, in the intent-to-treat sample (N = 73), 18 participants (51.4%) in the MST group (N = 35) and 16 (42.1%) in the ECT group (N = 38) met response criteria, while 13 (37.1%) in the MST group and 10 (26.3%) in the ECT group met remission criteria. In addition to the two experimental conditions (MST and ECT), covariates including age, sex, duration of illness, age of onset, intelligence quotient (IQ), and antidepressant usage were considered, resulting in a total of 6 covariates. Accounting for a potential dropout rate of 20%, the final estimated number of participants required for the study was determined to be 210. (**Figure 2**).

### Randomization procedure and blinding

A permuted block randomization method will be utilized to allocate participants to treatment groups at each study site. The sample size was consistent across sites, with 50% of participants assigned to receive ECT and the remaining 50% allocated to receive MST. Both clinical staff and participants were blinded to the treatment assignment. Randomization numbers were sealed in opaque envelopes, which were opened by the designated seizure therapy technician responsible for administering either ECT or MST treatment, based on the randomization numbers, only at the start of treatment. Randomization occurred on Day 1 immediately following baseline data collection. Following completion of the intervention, participants will be asked to provide feedback on whether they believe they received ECT or MST stimulation.

### Assessments

#### Clinical measures

Clinical assessments will be administered by blinded raters at various time points throughout the study, including baseline, post-session 3, 6, 9, and 12 (3 hours after each session), as well as at the 4-week, 8-week, and 12-week follow-up visits. The primary outcome measure was the total score on the 24-item Hamilton Depression Rating Scale (HDRS-24) (28). Additionally, the Hamilton Anxiety Scale (HAMA) (30), clinician-rated, served as a secondary outcome measure. Other assessments included self-reported measures such as the Self-Rating Depression Scale (SDS) (31) and the Self-Rating Anxiety Scale (SAS) (32), as well as clinician-rated measures including the Global Assessment of Functioning (GAF) scale (33). Follow-up assessments were conducted monthly. Prior to the commencement of the experiment, all subjects will undergo an interview using the MINI-International Neuropsychiatric Interview (M.I.N.I. 5.0) (34). Additionally, participants filled out a self-administered questionnaire to provide demographic information.

#### Cognitive assessments

The assessors conducting cognitive tests will be blinded to the group allocation of the patients. Moreover, the timing of the cognitive assessments will coincide with the time points of clinical evaluations. The WASI will be utilized to evaluate intellectual function. Consisting of four subtests, the WASI assesses two verbal aspects of crystallized intelligence (vocabulary and similarities) and two nonverbal measures of fluid intelligence (block design and matrix reasoning). While the WASI subtests share similarities with those found in the Wechsler Adult Intelligence Scale – Third Edition, they feature distinct items. Cognitive function will be assessed in the study using a battery of eight tests, with the Hopkins Verbal Learning Test-Revised (HVLT-R) designated as the primary cognitive outcome (evaluating verbal learning and memory). The remaining seven tests serve as secondary cognitive outcomes: Trail Making Test Part A (TMT-A), Brief Assessment of Cognition in Schizophrenia, Symbol Coding (BACS SC), Category Fluency Test, Continuous Performance Test-Identical Pairs version (CPT-IP), Wechsler Memory Scale, Third Edition, Spatial Span Test (WMS-III SS), Brief Visuospatial Memory Test, Revised Edition (BVMT-R), and Neuropsychological Assessment Battery: Mazes Test (NAB Mazes). These tests encompass various cognitive domains such as processing speed, attention, verbal and visual learning, reasoning, problem-solving, and working memory. Test-retest reliability in a prior Chinese sample ranged from 0.73 to 0.94 (35).

#### EEG acquisition

Participants will be seated in a dimly lit, sound-attenuated, and electrically shielded room for EEG data collection. EEG data will be recorded using a 64-channel electrode cap (Brain Products Inc., Bavaria, Germany). Electrodes for electrooculography (EOG) will be positioned below the left eye and above the right eyebrow. Bilateral mastoids will be utilized as reference electrodes, with AFz serving as the ground electrode. EEG signals will be digitized at a sampling rate of 1000 Hz and bandpass filtered between 0.016 and 200 Hz using filters from Brain Products Inc. Electrode impedance will be maintained below 5 kΩ throughout EEG data collection. Participants will be instructed to sit comfortably with their eyes closed for 5 minutes and then with their eyes open for another 5 minutes.

Offline preprocessing and subsequent analysis of resting state EEG (rsEEG) data will be conducted using EEGLAB (36) and MATLAB (MathWorks, Natick, Massachusetts). The EEG data preprocessing pipeline will involve several steps to ensure data quality and prepare it for analysis. This included bandpass filtering between 0.5 and 70 Hz with notch filtering at 50 Hz, downsampling to 512 Hz, and visual inspection to remove epochs with severe artifacts. Independent component analysis (ICA) will be used to remove blink and horizontal eye movement artifacts, followed by interpolation of bad channels and re-referencing to the average reference. Subsequently, artifact-free segments of 60 seconds will be selected for power spectral density estimation using Welch’s method with a Hamming window of 2 seconds and 50% overlap between segments, resulting in a frequency resolution of 0.5 Hz (37). Absolute power values will be calculated for predefined frequency bands, including delta (1–4 Hz), theta (4–8 Hz), alpha1 (8–10 Hz), alpha2 (10-12.5 Hz), beta1 (12.5-18.5 Hz), and beta2 (18.5–30 Hz). Additionally, relative power will be computed as the proportion of power within each specific frequency band relative to the total power across all frequency bands. Analyses of EEG data are exploratory and aimed at identifying potential associations with treatment outcomes.

#### ECG acquisition

All participants will undergo 10-minute resting ECG monitoring concurrent with the EEG recording. Participants will be seated comfortably in a quiet room and instructed to maintain even breathing, avoid body movements, and refrain from talking. ECG signals will be recorded using a single-channel system with three electrodes: the red electrode placed on the right forearm, the black electrode on the left forearm, and the yellow electrode on the left shank. Data will be collected using a portable electronic analyzer and computerized analysis system (Heart Rate Analyzer HW6C, Medeia Co., Ltd., Santa Barbara, CA, USA) at a sampling rate of 1000 Hz.

Subsequently, ECG signals will undergo preprocessing to identify artifacts and will be transformed into R-R inter-beat interval (RRI) time series using the NeuroKit2, a standardized neurophysiological signal processing toolbox developed in Python. The toolbox will analyze and extract HRV indices from the RRI series. In this study, utilizing 5-minute ultra-short-term ECG recordings, heart rate and a total of 14 HRV indices will be extracted and included in the analyses:

1. HR (bpm): Mean heart rate times per minute

Time-domain HRV Indices

2. SDNN (ms): Standard deviation of the RRI series, a representative variable in time-domain analysis influenced by both sympathetic and parasympathetic activity
3. SDNNI (ms): SDNN index, mean of the standard deviation of RRIs extracted from 1-minute segments of time series data
4. RMSSD (ms): Root mean square of successive differences between RRIs, measuring vagal contribution to HRV without being affected by respiration
5. pNN50 (%): Percentage of RRIs greater than 50ms out of the total number of RRIs, reflecting PNS activity
6. HTI: HRV triangular index, a geometric measure calculating the total number of RR intervals divided by the height of the RR intervals histogram, reflecting arrhythmias jointly with RMSSD, and less affected by noise and artifacts

Frequency-domain HRV Indices

7. TP (ms²): Total power of the density spectral
8. LF (ms²): Absolute power of the low frequency (0.04-0.15 Hz) band, primarily reflecting SNS activity
9. HF (ms²): Absolute power of the high frequency (0.15-0.40 Hz) band, reflecting parasympathetic nervous activity and periodicity of respiration
10. LF/HF: Ratio of LF power over HF power, an index of sympathovagal balance measuring the relative contributions of SNS to PNS activity
11. LFn: Normalized LF power; LFn = LF/(LF+HF);
12. HFn: Normalized HF power; HFn = HF/(LF+HF); LFn and HFn have been demonstrated to be less sensitive to the changes in total power

Nonlinear HRV Indices

Nonlinear indices of HRV in this study will be derived from Poincaré plot analysis, a geometrical and nonlinear technique of phase-space characterization. The Poincaré plot is a scatter plot graphed by plotting every RRI against the prior interval and we can analyze it by fitting an ellipse to the points. This method provides a visual summary of heart behavior patterns buried in a time series.

13. S (ms): Area of ellipse, representing total HRV
14. SD1 (ms): Standard deviation of the distance of each point from the y = x, measuring short-term HRV and correlated with baroreflex sensitivity (BRS)
15. SD2 (ms): Standard deviation of each point from the y = x + average R–R interval, measuring both short-term and long-term HRV

Analyses of ECG-derived HRV indices are exploratory, with the goal of examining their potential relationships with treatment response and cognitive side effects.

### Outcomes

#### Primary outcome

The primary outcome measure is the change in HDRS-24 total score from baseline to the 12-week follow-up, which is assessed after the completion of the 12-session acute treatment phase (conducted over 4 weeks) and a subsequent 12-week post-treatment observation period. A reduction of at least 50% in the HDRS-24 score indicated a response to treatment, while a reduction of at least 60% in the HDRS-24 score and a total score of 8 or less indicated remission. Change in HVLT-R total recall score from baseline to the 12-week follow-up (assessing verbal memory, a key cognitive domain sensitive to seizure therapy effects).

#### Secondary outcomes

The secondary outcomes include: changes in HAMA, SDS, SAS, and GAF scores over time; changes in the seven secondary cognitive variables (TMT-A, BACS SC, Category Fluency Test, CPT-IP, WMS-III SS, BVMT-R, NAB Mazes); and changes in rsEEG and ECG HRV variables.

Relapse is defined as an HDRS-24 total score of 16 or higher, with at least a 10-point increase from the lowest post-treatment score, sustained across two visits at least 1 week apart. If rescue treatment (e.g., maintenance ECT, adjusted pharmacotherapy) is administered before Week 12, outcomes will be recorded up to the point of intervention to assess time-to-relapse.

### Intervention

Patients will receive either MST, administered at a frequency of 100 Hz with 100% of the maximum device power for a duration of 10 seconds, or bitemporal ECT, according to routine clinical practice at SMHC following the ECT clinical practice guideline in China. The electrical stimulation wave width will be 0.5 ms. The energy of electrical stimulation will be determined according to the patients’ age(age × 0.8 × 100%) and increased by 5% until a proper seizure (seizure duration of 15 seconds or longer) is achieved.

### Anesthesia

The MST/ECT procedure will be conducted under general anesthesia, which will include intravenous administration of etomidate (0.21–0.3 mg/kg) and propofol (1.82–2.44 mg/kg). Intravenous succinylcholine (1 mg/kg) will be administered as a muscle relaxant, while intravenous atropine (0.5 mg) will be administered to reduce airway secretions.

### ECT and MST procedures

For the ECT group, a Thymatron System IV device (Somatics LLC, USA) will be used with bilateral (BL) electrode placement. This configuration is selected because it represents the most commonly adopted ECT modality in majority of psychiatric hospitals in China. The MST will be delivered with NS 7000S (Wuhan Yiruide Medical Equipment New Technology Co., Ltd.) using a round coil (125-mm diameter) positioned on the vertex. At 100% output and 100 Hz frequency, the device generates a peak magnetic field strength of 3.0 T and a pulse width of 350 μs. Compared to the MagPro MST system, which uses a dual-coil design with 2.0 T surface strength and 370 μs biphasic pulses, the YIRUIDE NS 7000s offers higher peak field strength and slightly shorter pulse width, potentially enhancing seizure induction efficiency in preclinical practice.

For ECT, the pulse width of the electrical stimulus will be set to 0.5 ms. Seizure threshold titration is the primary method for determining dosage: at the first treatment session, the psychiatrist and anesthetist will administer gradually increasing stimuli until the minimum charge required to induce a 25-second EEG seizure is identified (titration method). This threshold guides the initial supra-threshold dosage (1.5× threshold) for subsequent sessions. As a secondary reference in line with local clinical practice, the initial energy dosage may also be estimated using an age-based formula (age × 0.8 × 100%) for the first session, but this is adjusted based on titration results. For subsequent sessions, dosage will be increased by 5% increments if seizure duration is inadequate (<25 s); in cases of persistent suboptimal seizures, the maximum tolerated dosage will be administered. This approach prioritizes individualized titration to balance efficacy and safety, while exploring the utility of age-based estimates for resource-constrained settings. Additionally, we will monitor data to explore the empirical ‘half-age’ method (38) for future reference.

MST will be conducted at 100 Hz and 100% output using a pulse width of 350 μs and a peak intensity of the magnetic field at 3.0 Tesla. The duration of magnetic stimulation will be titrated using a 10-second train duration, starting from 2 seconds and increasing by 4 seconds in subsequent sessions, up to a maximum of 10 seconds (i.e., 200–1,000 pulses per session). In cases of poor seizure quality (<15 s), the stimulation duration increment will be set to 6 seconds in the next session. If no seizures were elicited, an additional 10-second stimulation will be administered immediately. Subsequent MST treatments will be maintained at a 10-second duration for a total of 1000 pulses.

EEG recordings during MST and ECT will be obtained using the Thymatron IV device with left and right frontal leads.

Patients will undergo identical preparation procedures, including the placement of earplugs and skin preparation for ECT electrode placement, irrespective of treatment allocation. To maintain blinding, the auditory stimulus from a recorded MST session will be played during ECT administration to prevent treatment allocation disclosure to individuals in adjacent rooms.

Completers will be defined as patients who received at least 8 treatments or achieved remission before the eighth treatment. Relapse will be defined as an HDRS-24 total score of 16 or higher, with at least a 10-point increase in HDRS-24 score sustained across two visits at least 1 week apart, the emergence of psychotic or suicidal symptoms, or the need for readmission to inpatient care.

While 12 sessions are standard for acute-phase treatment, the potential need for more sessions in MST will be explored through *post-hoc* analyses. This trial’s design prioritizes comparability with ECT’s typical acute regimen while allowing for long-term outcome assessment to capture delayed benefits.

### Patient safety

We implement proactive site monitoring and adhere to established safety protocols as standard procedures in our previous ECT/MST studies (26, 27). Electrophysiological recordings pose no known risks, and there is no evidence of short- or long-term side effects associated with them. However, a small proportion of participants may experience scalp discomfort during the procedure. In the afternoon following each treatment session, patients will be interviewed using the Columbia ECT Subjective Side Effects Schedule (39) to assess subjective adverse effects. Patients will be asked to report the presence (scored as 0 for absent and 1 for present) and severity (scored as 0 for no perceived adverse effect, 1 for mild, 2 for moderate, and 3 for severe) of various adverse effects. Additionally, any potential severe adverse events will be meticulously documented by the experimenter.

### Additional treatments

As the primary objective of this trial is to evaluate efficacy, participants are restricted from receiving concurrent treatments for their depressive symptoms, except for one type of antidepressant medication, during the 30-day intervention period. However, the use of psychoactive medications and psychotherapy is allowed during the 12-week follow-up period, with detailed documentation of the type, dosage, and frequency/session of medication or psychotherapy.

During the 12-week follow-up period, participants may receive maintenance ECT, pharmacotherapy (e.g., antidepressants, mood stabilizers), or psychotherapy to prevent relapse. The type, dosage, and frequency of maintenance treatments will be recorded in detail to assess their impact on outcomes.

### Withdrawal criteria

Criteria for participant withdrawal from the study comprise the following: 1) Failure to complete a minimum of 8 ECT/MST treatment sessions within the designated 30-day period. Participants missing scheduled sessions may reschedule them at their convenience, but failure to complete the required sessions will result in dropout status. 2) Voluntary withdrawal of consent for ECT/MST treatment by the participant. 3) Experience of a severe adverse event with significant distressing consequences, such as seizures, skin burns, or blisters. Withdrawn participants will continue to be monitored, and outcome measures will be conducted to the extent possible.

## Data entry and analyses

This trial aims to provide replicated evidence that MST is as effective as ECT in terms of remission rates, response rates, and reduction of depressive symptoms (assessed by the primary outcome: change in HDRS-24 from baseline to 12-week follow-up after the acute treatment phase) and superior in preserving verbal memory (via HVLT-R as an additional primary outcome), with fewer physical and cognitive side effects overall. Additionally, this study will explore personalized electrophysiological indicators (EEG/ECG) in an exploratory manner to inform future individualized treatment decisions for ECT and MST, and to generate hypotheses about the physiological mechanisms underlying the antidepressant efficacy and cognitive effects of MST/ECT. To our knowledge, this will be the largest RCT comparing ECT and MST in China. The trial will provide evidence to determine whether individualized treatment decisions based on personalized electrophysiological characteristics hold promise for optimizing the balance between efficacy and side effects (40). Specifically, it will offer insights into the potential effect sizes, acceptability, feasibility, and safety of the novel interventions under investigation. Predicting efficacy and side effects based on physiological characteristics, such as indicating greater side effects with ECT and comparable efficacy but fewer side effects with MST for patients with MDD, suggests that MST may be more suitable for these patients. Alternatively, physiological characteristics indicating superior efficacy with ECT compared to MST, with comparable side effects between the two, suggest that ECT may be more appropriate for these patients.

Firstly, data entry will undergo a thorough double-check to ensure accuracy. Data quality will then be assessed, with particular attention given to data distribution, and outliers will be excluded from subsequent analysis. Once data integrity is confirmed, detailed analyses of primary and secondary outcomes will be conducted using IBM SPSS Statistics 20 for Windows (Armonk, NY: IBM Corp).

Descriptive statistics, such as mean and standard deviation, will be computed for each study arm separately. Analyses will be performed within the intention-to-treat (ITT) framework to assess the effectiveness of interventions for all participants, utilizing multiple imputed datasets to address missing data.

Appropriate statistical methods will be employed for data analysis, including mixed-effects models for repeated measures (MMRM). MMRM with a factorial design (3 time points × 2 conditions) will be conducted to examine significant interactions between time (baseline, Day 30, and Week 12) and condition (ECT and MST), while adjusting for potential covariates such as age, sex, duration of illness, age of onset, IQ, and antidepressant usage. This approach is robust to missing data under the missing-at-random (MAR) assumption and provides unbiased estimates of treatment effects. The significance level will be set at 0.05.

Sensitivity analyses will be performed to assess the robustness of results to missing data, including multiple imputation (MI) under MAR and missing-not-at-random (MNAR) assumptions. Results from MMRM and MI will be compared. Exploratory analyses will include subgroup analyses by demographic/clinical characteristics and correlation analyses between neurophysiological biomarkers (EEG/ECG) and treatment outcomes, using linear or logistic regression models as appropriate.

Subgroup analyses will compare relapse rates and time-to-relapse between participants who received maintenance treatments versus those who did not, within both MST and ECT groups. This will help disentangle the effects of acute treatment from post-treatment management strategies.

## Discussion

Our study design features several strengths. Firstly, we have enrolled a sizable sample size and meticulously documented participants’ electrophysiological characteristics throughout the entire ECT/MST treatment process and the subsequent follow-up period. This comprehensive approach adds significant value to our study by providing a detailed understanding of the effects of these therapies. Secondly, we have implemented a strict blindng protocol, a challenging aspect in ECT and MST studies. To ensure the quality of blinding, third-party evaluators have been invited for assessment. Moreover, patients undergo identical preparation for both ECT and MST treatments, and the MST device emits similar sounds during sessions to maintain blinding. Thirdly, we have made efforts to control confounding factors by allowing only a single stable dose of antidepressant medication during the treatment process. Fourthly, while previous comparisons between ECT and MST often view MST as a mere replacement for ECT, our study aims to optimize treatment decisions through personalized electrophysiological signals. Lastly, there is currently a lack of efficacy prediction models based on EEG and ECG physiology in seizure therapy (41, 42). If our study can establish an effective model, it will provide a novel tool for guiding precision seizure therapy.

However, our protocol is not devoid of risks and potential limitations, which we strive to address. Firstly, depression is a chronic and fluctuating condition, and the 12 sessions of stimulation and 12-week follow-up planned for our patients may not be adequate to induce or fully capture significant improvements. While a more intensive regimen with increased cumulative sessions over time and longer follow-up duration would be beneficial, it is currently beyond the scope of our protocol due to constraints on time and resources. Secondly, individuals with MDD, despite sharing similar depressive symptoms, may be influenced differently by various biological factors such as inflammation, genetic susceptibility, or aberrant brain function, which can impact the effectiveness of ECT/MST treatment. Although our methods incorporate objective measures of EEG and ECG physiology, they only represent a fraction of the etiology of MDD and cannot encompass all biological subtypes of MDD. Lastly, the neurobiological mechanisms underlying the effects of ECT/MST on MDD remain largely unclear. While our study includes the collection of EEG and ECG signals to investigate the electrophysiological mechanisms of seizure therapy, these assessments are conducted offline and can only provide limited insights into the mechanisms of action. Future studies could consider additional assessments, such as brain functional characteristics based on magnetic resonance imaging (43, 44), to further elucidate the neural mechanisms of seizure therapy on brain function.

One notable limitation is the use of an age-based dosing method for ECT (80% of the patient’s age in years × 100 mC), which is less precise than seizure threshold titration. This approach may lead to suboptimal dosing in some patients, such as underdosing in younger individuals with higher seizure thresholds or overdosing in older adults with lower thresholds, potentially increasing the risk of cognitive side effects or reducing treatment efficacy. The decision to use age-based dosing reflects common clinical practice in many Chinese centers, where titration protocols are not yet universally adopted due to resource constraints or training variability. While this aligns with real-world settings, it introduces variability in ECT dosing that could affect the comparability of safety and efficacy outcomes with MST. Future studies using more personalized dosing strategies (e.g., titration) are needed to validate the generalizability of our findings. An additional limitation is the absence of a dedicated autobiographical memory test in our cognitive assessment battery. Autobiographical memory is a domain frequently reported to be sensitive to ECT-mediated cognitive impairments, and its exclusion may limit our ability to fully capture differences in this specific memory function between MST and ECT. Future studies are encouraged to incorporate validated autobiographical memory measures to complement assessments of verbal learning and memory and provide a more comprehensive evaluation of cognitive effects.
